# LC-ESI-QTOF/MS Characterisation of Phenolic Acids and Flavonoids in Polyphenol-Rich Fruits and Vegetables and Their Potential Antioxidant Activities

**DOI:** 10.3390/antiox8090405

**Published:** 2019-09-17

**Authors:** Chunhe Gu, Kate Howell, Frank R. Dunshea, Hafiz A. R. Suleria

**Affiliations:** School of Agriculture and Food, Faculty of Veterinary and Agricultural Sciences, The University of Melbourne, Parkville 3010, VIC, Australia; chunheg@student.unimelb.edu.au (C.G.); khowell@unimelb.edu.au (K.H.);

**Keywords:** Polyphenols, fruits and vegetables, antioxidant activities, phenolic acids, flavonoids, HPLC-PDA, LC-ESI-QTOF/MS

## Abstract

Polyphenols are naturally occurring compounds found largely in fruits and vegetables. The antioxidant properties of these polyphenols including total phenolic content (TPC), total flavonoid content (TFC), tannin content, 1,1-diphenyl-2-picrylhydrazyl free radical (DPPH), 2,2′-azinobis-(3-ethylbenzo-thiazoline-6-sulfonic acid) (ABTS) scavenging abilities and ferric ion reducing antioxidant power (FRAP) were measured among sixteen (16) plant foods (mango, blueberry, strawberry, black carrot, raspberry, dark grapes, garlic, ginger, onion, cherry, plum, apple, papaya, peach, pear and apricot) by modifying, standardising and translating existing antioxidant methods using a 96-well plate reader. Eighteen targeted phenolic acids and flavonoids were characterised and quantified using high-performance liquid chromatography-photometric diode array (HPLC-PDA) and verified by modifying an existing method of liquid chromatography coupled with electrospray-ionisation triple quadrupole time-of-flight mass spectrometry (LC-ESI-QTOF/MS). While most of these compounds were accurately detected by the HPLC-PDA at a low concentration, a few polyphenols in low concentrations could be only be characterised using the LC-ESI-QTOF/MS method. Our results showed that mango possessed the highest overall antioxidant activity, phenolic acid and flavonoid content among the selected fruits. Factor analysis (FA) and Pearson’s correlation tests showed high correlations among ABTS, DPPH, FRAP and phenolic acids, implying the comparable capabilities of scavenging the DPPH/ABTS free radicals and reducing ferric ions from the antioxidant compounds in the samples. Phenolic acids contributed significantly to the antioxidant activities, and flavonoids contributed more to tannin content based on the correlations. Overall, methods modified and standardized in this study can provide better understanding of high throughput technologies and increase the reliability of antioxidant data of different plant foods.

## 1. Introduction

The importance of polyphenols as bioactive compounds is widely accepted, and polyphenols are used as additives in food, feed, nutraceutical and pharmaceuticals industries. Polyphenols are beneficial to human and animal health because of their antioxidant activities [1]. Epidemiological evidence shows that polyphenols prevent the generation of free radicals and reactive oxygen species (ROS) during metabolism and are thus associated with reduced risk of chronic diseases caused by excessive oxidative stress such as atherosclerosis, inflammation and different types of cancers [2,3]. Beyond the role in physiological systems, polyphenols can be used in the food industry to prolong the shelf life of manufactured products and to replace synthetic antioxidants often used for these purposes [4]. Classes of polyphenols are categorised based on diverse chemical structures, which is the decisive factor of antioxidant activities.

Phenolic acids are primary components of polyphenols in plant foods. Phenolic acids are associated with anti-carcinogenic and anti-inflammatory activities because of the radical-scavenging ability [5]. Similarly, flavonoids are a subclass of polyphenols widespread in fruits and vegetables with some responsible for the colours of the plants [6]. Particularly, tannins are flavonoids prevalent in plant foods that causes sensory property of astringency. Other than antioxidant activities, epidemiological evidence also suggests that flavonoids are potential therapeutics for Alzheimer’s disease due to the ability of attenuating amyloid-β oligomer-induced neuronal responses as antioxidants [7].

Antioxidant activities of polyphenolic mixtures are evaluated using different in vitro spectrophotometric-based assays such as total phenolic content (TPC), total flavonoid content (TFC), radical scavenging activity using 1,1-Diphenyl-2-picryl-hydrazyl (DPPH) assay and 2,2′-azinobis-(3-ethylbenzothiazoline-6-sulfonic acid) (ABTS) assay, reducing ability using ferric reducing antioxidant potential (FRAP) assay, and assays for specified tannin polyphenols. Many studies are carried out using cuvettes and test tubes with high solvent volumes and are quite laborious [8,9,10]. Performing antioxidant assays in 96-well plate is quick, efficient and reduces the volume of reagents and solvents. Previously, a few methods were published to optimise antioxidant assays (DPPH and ABTS) using 96-well plate reader [11], high-throughput rapid antioxidant assay (TPC) for the quantification of reducing capacity of foods [12] and detection of online antioxidant activity (DPPH and FRAP) [13], but all of these methods are not mapping an overall phytochemistry and their potential antioxidant activities. Therefore, developing standardised methods to evaluate overall antioxidant activities and screening of targeted polyphenols from different fruits and vegetables using high pressure liquid chromatography coupled with mass spectrometry was of interest.

The aim of our study was to modify, standardise and translate existing methods for antioxidant assays (TPC, TFC, tannins content, DPPH, FRAP and ABTS), verify their sensitivity, reproducibility and accuracy using a 96-well plate UV-Vis spectrophotometer. One of our main targets was to modify existing chromatographic methods using a combination of high-performance liquid chromatography-photometric diode array (HPLC-PDA) and liquid chromatography coupled with electrospray-ionisation triple quadrupole time-of-flight mass spectrometry (LC-ESI-QTOF/MS) for identification and characterisation of 18 targeted polyphenols (9 phenolic acids and 9 flavonoids) that commonly exist in plant foods with high accuracy. The standardised high-throughput mode and verification of the chromatography analyses are expected to help better understand the analytical technologies most appropriate to detect the antioxidant compounds of plant foods.

## 2. Materials and Methods

### 2.1. Chemicals and Reagents

All chemicals were analytical grade or analytical standards and were purchased from Sigma-Aldrich (Castle Hill, NSW, Australia). Analytical grade methanol, ethanol and formic acid were used for polyphenol extraction. Folin-Ciocalteu reagent, sodium carbonate, gallic acid, aluminium chloride, sodium acetate, quercetin, vanillin, sulfuric acid, catechin, 2,2-Diphenyl-1-pricrylhydrazyl (DPPH), ascorbic acid, sodium acetate, 2, 4, 6-tripyridyl-s-triazine (TPTZ), ferric chloride, ABTS and potassium persulfate were used for antioxidant assays. 96-well plates (flat bottoms with 300 µL total volume) (Corning Inc., Corning, NY, USA) were purchased from Thermo Fisher Scientific (Scoresby, VIC, Australia). HPLC analytical grade gallic acid, protocatechuic acid, caftaric acid, p-hydroxybenzoic acid, catechin, chlorogenic acid, caffeic acid, syringic acid, epicatechin, coumaric acid, epicatechin gallate, ferulic acid, quercetin-3-*O*-glucuronide, quercetin-3-*O*-galactoside, kaempferol-3-*O*-glucoside, quercetin, kaempferol, acetic acid and acetonitrile were for chromatographic analysis. The 1.5 mL HPLC vials were purchased from Agilent Technologies (Mulgrave, VIC, Australia). 

### 2.2. Antioxidant Assays

#### 2.2.1. Samples Preparation

Sixteen polyphenol-rich fruits and vegetables, including mango (*Mangifera indica*), blueberry (*Cyanococcus*), strawberry (*Fragaria × ananassa*), black carrot (*Daucus carota sativus var. atrorubens*), raspberry (*Rubus idaeus*), dark grapes (*Vitis vinifera*), garlic (*Allium sativum*), ginger (*Zingiber officinale*), onion (*Allium cepa*), cherry (*Prunus avium*), plum (*Prunus subg. Prunus*), apple (*Malus domestica*), papaya (*Carica papaya*), peach (*Prunus persica*), pear (*Pyrus*) and apricot (*Prunus armeniaca*) were purchased from a local market in Melbourne, Victoria. All the fruit and vegetable samples used in this study were fully matured, ripened and grown at different parts of Victoria, Australia. The edible portion of the plant samples were chopped into cubes, 2–3 kg of each sample was weighed and blended into a slurry using a 1.5 L blender (Russell Hobbs Classic, model DZ-1613, Australia). A small amount of Milli Q water was added before blending if the sample was too dry. Polyphenols were extracted with 30% ethanol at a ratio of 0.1 g slurry/mL. After weighing the samples and adding the extraction solvent to the centrifuge tubes, the samples were then homogenised at 5,000 rpm for 2 min using an IKA Ultra-Turrax^®^ T25 homogenizer (Rawang, Selangor, Malaysia). The extraction was taken in a shaking incubator (ZWYR-240, Labwit, Ashwood, Australia) at 120 rpm 4 °C for 12 h in darkness. Afterwards, the tubes were centrifuged at × 10,000 *g* for 10 min. The supernatant was collected and diluted with ethanol at appropriate ratios for the various antioxidant analysis. 

#### 2.2.2. Antioxidant Assays 

All the antioxidant assays were modified and translated into 96-well plates based on the methods in previous literature reports. The data was measured by a Multiskan FC microplate photometer (Thermo Fisher Scientific, Australia). All tests were run in triplicate. The standard curves were created with R^2^ > 0.995. 

##### Total Phenolics Content (TPC) Assay

The TPC assay was adapted from the method described by Singleton and Rossi [14] with modifications. Here, 25 µL sample was mixed with 25 µL Folin’s reagent which was diluted 3 times with water in a 96-well plate. Then 200 µL water was added, and the mixture was incubated at room temperature for 5 min. Afterwards, the mixture was mixed with 25 µL 10% (w:w) sodium carbonate and incubated at 25 °C for 60 min. The absorbance was measured at 765 nm. Concentrations of 0 to 200 µg/mL gallic acid dissolved in ethanol were made to construct the standard curve. The results were expressed as mg gallic acid equivalents (GAE) per g of fresh sample weight.

##### Total Flavonoid Content (TFC) Assay 

The TFC assay was initially carried out by Christ and Müller [15] and was modified in subsequent reports. Our method was adapted from Horszwald, et al. [16] with sodium acetate as medium and quercetin as the standard compound. Briefly, 80 µL of the samples was mixed with 80 µL 2% aluminium chloride diluted in ethanol followed by adding 120 µL of a 50 g/L sodium acetate solution. The mixture was incubated at 25 °C for 2.5 h and the absorbance was measured at 440 nm. Concentrations of 0 to 50 µg/mL quercetin dissolved in ethanol were made to construct the standard curve. The results were expressed as mg quercetin equivalents (QE) per g of fresh sample weight.

##### Tannin Assay

The tannin assay was based on a modified version from Price, et al. [17]. Here, 25 µL of sample solution was added by 150 µL 4% vanillin solution and then mixed with 25 µL sulfuric acid. Both vanillin and sulfuric acid were diluted with ethanol. The mixture was incubated at 25 °C for 15 min and the absorbance was measured at 500 nm. Concentrations of 0 to 1000 µg/mL catechin dissolved in ethanol were made to construct the standard curve. The results were expressed as mg catechin equivalents (CE) per g of fresh sample weight.

##### 1,1-Diphenyl-2-picryl-hydrazyl (DPPH) Assay

The radical scavenging ability of DPPH assay was modified from the method reported by Mensor, et al. [18]. Briefly, 0.1 mmol/L DPPH solution was firstly prepared in methanol. We added 260 µL of the DPPH solution was added by 40 µL sample. The mixture was incubated for 30 min at 25 °C. Then the absorbance was measured at 517 nm. Concentrations of 0 to 50 µg/mL ascorbic acid dissolved in water were used to make the standard curve. The results were expressed as mg ascorbic acid equivalents (AAE) per g of fresh sample weight.

##### 2,2′-azinobis-(3-ethylbenzothiazoline-6-sulfonic acid) (ABTS) Assay

The ABTS assay followed the procedure described by Re, et al. [19] with modifications. Here, 5 mL of 7 mmol/L of ABTS solution was mixed with 88 µL of a 140 mM potassium persulfate solution to produce ABTS^+^. The mixture was placed in the dark at room temperature for 16 hours. Then 0.5 mL of the ABTS^+^ solution was diluted by adding 45 mL ethanol. The absorbance was checked at 734 nm with stable reading at 0.7. The sample extracts (10 µL) were then taken and added to 290 µL prepared dye solution. The mixture was then incubated at 25 °C for 6 min, and the absorbance was measured at 734 nm. Concentrations of 0 to 150 µg/mL ascorbic acid dissolved in water were made to construct the standard curve. The results were expressed as mg ascorbic acid equivalents (AAE) per g of fresh sample weight. 

##### Ferric Reducing Ability (FRAP) Assay

The FRAP assay was carried out to evaluate the reducing ability based on the method reported by Benzie and Strain [20]. A stock solution of 300 mmol/L sodium acetate buffer was added to 10 mmol/L 2,4,6-tripytidyl-s-triazine (TPTZ) solution, and 20 mmol/L ferric chloride at a ratio of 10:1:1 (v:v:v). Plant extracts (20 µL) were mixed with 280 µL dye solution and incubated at 37 °C for 10 min. The absorbance was then measured at 593 nm. Concentrations of 0 to 50 µg/mL ascorbic acid were made to construct the standard curve. The results were expressed as mg ascorbic acid equivalents (AAE) per g of fresh sample weight. 

### 2.3. HPLC-PDA Analysis 

The quantification of targeted phenolic compounds present in different fruits and plant samples were carried out by the HPLC (Waters Alliance 2690, Chromatograph Separation Module) equipped with a photodiode array (PDA) detector (Model 2998, Waters) according to the method of Schieber, et al. [21] with modifications. A Synergi Hydro-RP (250 × 4.6 mm i.d.) reversed phase column with a particle size of 4 µm (Phenomenex, Lane Cove, NSW, Australia) was protected by a Phenomenex 4.0 × 2.0 mm i.d., C18 ODS guard column. The mobile phase consisted of water/acetic acid (98:2, v/v; eluent A) and acetonitrile/water/acetic acid (100:1:99, v/v/v; eluent B). The gradient profile was 10–25% B (0–20 min), 25–35% B (20–30 min), 35–40% B (30–40 min), 40–55% B (40–70 min), 55–80% B (70–75 min), 80–90% B (75–77 min), 90–100% B (77–79 min), 100–10% B (79–82 min), isocratic 10% B (82–85 min). The flow rate was set at 0.8 mL/min. The column was operated at room temperature and the samples temperature were set at 10 °C. The PDA detector was set at λ 280, 320 and 370 nm simultaneously. 

The extracts were filtered using syringe filer (0.45 µm PVDF, Millipore, MA, USA) and put into the HPLC vials (Agilent Technologies, Mulgrave, VIC, Australia). A volume of 50 µL was injected for each standard or sample. Instrument control, data acquisition and chromatography processing were performed using Empower Software (2010).

### 2.4. LC-ESI-QTOF/MS Analysis 

LC-ESI-QTOF/MS analysis was performed on an Agilent 1200 series HPLC (Agilent Technologies, CA, USA) equipped with an Agilent 6520 Accurate-Mass Q-TOF LC/MS (Agilent Technologies, CA, USA). Peak identification was performed in both positive and negative modes. Nitrogen gas nebulization was set at 45 psi with a flow rate of 5 L/min at 300 °C and the sheath gas was set at 11 L/min at 250 °C. The capillary and nozzle voltage were set at 3.5 kV and 500 V respectively. A complete mass scan ranging from *m*/*z* 50 to 1300 was used. Instrument control, data acquisition and processing were performed using MassHunter workstation software (Qualitative Analysis, version B.03.01, Agilent). The same column and conditions described in HPLC-PDA analysis maintained except for sample injection volume of 6 µL. The LC-ESI-QTOF/MS identified compounds with more than 80 library identification score were further selected for characterisation and *m*/*z* verification. 

### 2.5. Statistical Analysis

All the analyses were performed in triplicate. The values were expressed as mean ± standard deviation (SD). Microsoft Excel software (Microsoft, USA) and Minitab^®^ 17 Statistical software (Minitab Inc., State College, PA, USA) were used for generation of graphics. One-way analysis of variance (ANOVA) was used for comparisons of the antioxidant parameters and the polyphenol contents between samples. Factor analysis (FA) and Pearson’s test were applied to the understand the correlation between the variables since the dimension of the variables are small. The Varimax method was used for the orthogonal transformations to the reduced factors to better identify the high and low correlations. XLSTAT^®^ was applied for the FA and correlation analysis. 

## 3. Results and Discussion

### 3.1. Antioxidant Assays 

#### 3.1.1. Polyphenol Estimation (TPC, TFC and Tannin Content)

Among the selected samples, mango and blueberry possessed the highest TPC with 2.13 ± 0.10 and 2.08 ± 0.06 mg gallic acid equivalent (GAE)/g, followed by strawberry, black carrot, raspberry and grapes (Table 1). It should be noted that Folin-Ciocalteu reagent was used in this assay, which was not specific to only polyphenols but also could react with any other reducing substances that could be oxidised by the Folin reagent [22]. Therefore, other compounds with antioxidant activities such as ascorbic acid, which is rich in most of the fruits, could also contribute to TPC values. Pear and apricot had the lowest TPC. Both mango and blueberry have been reported as polyphenol-rich plants. Mango is rich in xanthones and flavonols, and most of the phenolic compounds exist in peel [23], and blueberry rich in phenolic acids, flavonoids and anthocyanins [24]. Similar trends but with higher values in TPC were reported previously in Chile fruit samples (harvested from Santiago, Chile) like blueberry (4.75 ± 0.34 mg GAE/g) and strawberry (4.02 ± 0.14 mg GAE/g) compared to pear (0.99 ± 0.07 mg GAE/g) extracted with different solvent methanol [25]. These higher TPC values might be due to the difference of fruit grown at different climate and region also extracted with different solvent. 

Polyphenols with flavonoid structures can react with aluminium chloride and undergo Al-flavonoid complexation reactions to form a yellow solution, which immediately turns red in an alkaline condition. Similar to the results of the TPC, blueberry had the highest TFC of 0.46 ± 0.02 mg of quercetin equivalent (QE)/g of the samples (Table 1). Comparable TFC values with a range of 0.40 to 0.50 mg QE/g fresh blueberries have been reported with several cultivars including Berkeley, Blueray, Darrow, Misty etc. [26], although higher values have been reported in other cultivars like Ozarkblue (77.72 ± 3.13 mg QE/g) and Bluegold (84.01 ± 1.81 mg QE/G). Catechin is the most important flavonoid compound in blueberry [24]. Onion, cherry and mango had high TFC with small statistical differences between these foods. Onion is an important dietary source of quercetin [27,28], and cherry is a rich source of kaempferol and cyanidin [29]. 

In fruits and vegetables, tannins are mostly composed of proanthocyanidins, which are polymers of oligomeric flavonoids with mostly catechin and epicatechin. Blueberry and garlic had high tannin contents of the foods tested here, with 2.35 ± 0.49 and 1.09 ± 0.01 mg catechin equivalent (CE)/g of the samples (Table 1). Previously, a slightly higher tannin content was reported in different garlic and onion varieties tested with the same method [30,31]. Limited amounts of tannin were detected in ginger, strawberry, black carrot, grapes, mango, pear and onion, and no tannins were detected in the remainder of the samples. The small amount of tannins determined in the selected samples might be contributed by the narrow range of compounds targeted in the vanillin assay, where only specified flavanols and dihydrochalcones with single bond at the 2,3-position and free meta-oriented hydroxyl groups on the B ring can participate in the reaction [32]. 

#### 3.1.2. Antioxidant Activities (DPPH, ABTS and FRAP)

The DPPH assay is widely used for antioxidant activity determinations and is based on the spectrophotometer absorption decrease at 517 nm due to the reduction of DPPH radicals scavenged by the antioxidant compounds. As shown in Table 1, mango, raspberry and strawberry showed the highest DPPH free radical scavenging activities with 2.34 ± 0.00, 1.63 ± 0.02 and 1.58 ± 0.06 mg ascorbic acid equivalents/g of the samples. In comparison, peach, garlic, pear and apricot showed the lowest DPPH free radical scavenging activity of the 16 plant extracts. Similar to the principle of the DPPH method, ABTS can also form a stable free radical and decolourisation also occurs as antioxidants reduce the pre-formed ABTS^•+^ [33]. The ABTS assay (Table 1) showed high similarity with the results from the DPPH assay with highest antioxidant activities from mangos and strawberry with 3.05 ± 0.13 and 2.23 ± 0.17 mg AAE/g, while lowest activities from peach and apricot. Similar comparisons were tested among popular antioxidant-rich plant foods previously, and peach (ABTS 0.70 ± 0.08 mg AAE/g and DPPH 0.65 ± 0.09 mg AAE/g) and pear (ABTS 0.94 ± 0.09 mg AAE/g and DPPH 0.68 ± 0.09 mg AAE/g) showed lower ABT and DPPH compared to strawberry (ABTS 2.74 ± 0.22 mg AAE/g and DPPH 5.21 ± 0.39 mg AAE/g) [34].

The FRAP assay is based on the antioxidant capable of donating a single electron to the Fe^3+^-TPTZ complex would result in the reduction of the complex into the blue Fe^2+^-TPTZ complex with high absorbance at 593 nm [8]. Mango, strawberry, raspberry and blueberry showed the highest values of 3.20 ± 0.05, 2.41 ± 0.05, and 2.40 ± 0.09 mg ascorbic acid equivalents/g of the samples. Spices including ginger, onion and garlic were detected with the lowest FRAP values. Generally, the results of all the antioxidant assays showed similar trends with mango, raspberry, strawberry and blueberry were detected with the highest antioxidant capacities, while peach, pear and garlic showed the relatively low antioxidant abilities. Similar results regarding the FRAP comparisons were gathered in study of Proteggente, et al. [35], where strawberry and raspberry showed the highest FRAP values (expressed as Fe^2+^ equivalent/g fresh weight) compared to onion, pear, apple and peach.

### 3.2. Characterisation of Phenolic Acids and Flavonoids Using HPLC-PDA and LC-ESI-QTOF/MS

#### 3.2.1. Quantification of the Phenolic Compounds Using HPLC-PDA

Nine phenolic acids and nine flavonoids that widely exist in fruits and vegetables were selected for quantification (Table 2). The flavonoids were mainly flavonols and flavan-3-ols and their corresponding glycosylated forms. The targeted phenolics were identified and quantified using the HPLC-PDA by comparing the retention times with the standards. The individual polyphenol was quantified based on linear regression of external standards plotting peak area against concentration ranges from 0 to 500 µg/mL (Supplementary file, Figure S1). The content of each phenolic compound was expressed as mg per 100 g fresh basis ± standard deviation, and the total phenolic acids and flavonoids were calculated by summarizing the individual phenolic acids and flavonoids together respectively (Table 2). The phenolic acids that existed in mango and blueberry were significantly higher than other fruits and vegetables with 139 ± 23.0 and 66.6 ± 20.18 mg/100 g fresh basis. Especially, gallic acid, protocatechuic acid and chlorogenic acid were the major phenolic acids in mangos, which is consistent with previous studies [23,36]. For flavonoids, the flavonoids in blueberry were 107 ± 26.4 mg/100g fresh basis with a high catechin content of 81.8 mg/100 g sample, and agrees with results presented by Haytowitz, et al. [37]. The flavonoids in mango were relatively low with a 19.1 ± 2.58 mg/100 g sample, reflecting that most flavonoid compounds exist in mango peel rather than the edible pulp analysed here [23]. Ginger also showed a very high flavonoid content with 57.4 ± 19.04 mg/100 g sample, in agreeance with previous data [38]. The HPLC results showed that comparing to other plant foods, spices including ginger, garlic and onion presented relative high flavonoids and limited phenolic acids, and reflected that quercetin and kaempferol are important biologically active compounds in spices [27]. 

#### 3.2.2. Identification and Characterisation of the Phenolic Compounds Using LC-ESI-QTOF/MS

The phenolic acids and flavonoids were furtherly characterised on mass basis using LC-ESI-QTOF/MS. The characterisation was carried out using both negative and positive modes, and better fragments were gathered with negative mode on for all the proposed polyphenols (Supplementary file, Figure S2). Table 3 shows the theoretical and observed *m*/*z* based on one example plant sample for each phenolic compound with mass errors within ± 10 ppm and library identification scores more than 80. 

Most of the polyphenols were accurately detected in HPLC-PDA. Nevertheless, some phenolic compounds were only detected by the LC-ESI-QTOF/MS but not the HPLC-PDA due to their low concentrations present in the samples. The omitted polyphenols from the HPLC-PDA detection are listed in Table 4, among which p-coumaric acid, p-hydroxybenzoic acid, protocatechuic acid, ferulic acid and caffeic acid were common compounds. This observation reflects that HPLC-PDA quantification can mis-identify compounds present in low concentrations, and thus results which only use HPLC-PDA should be used with caution. 

### 3.3. Correlations of Antioxidant Assays and Phenolic Compounds

**Figure 1:**
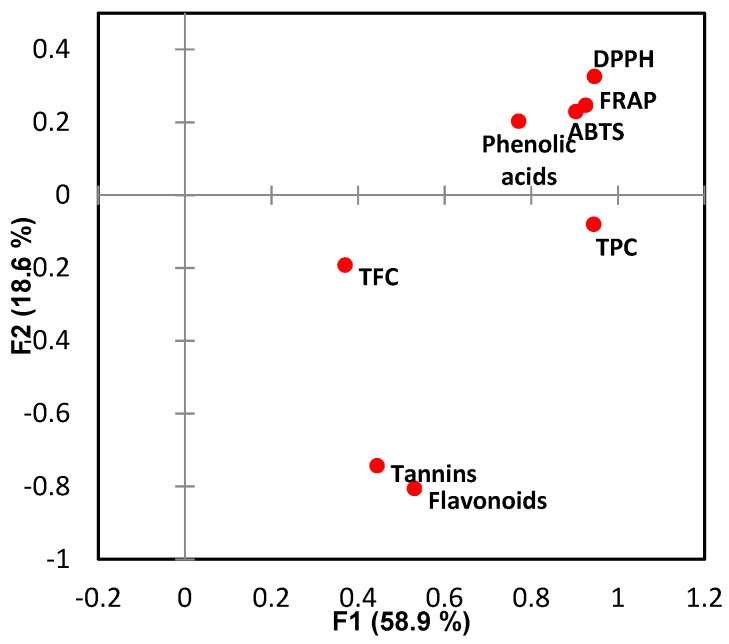
Factor analysis (FA) of the antioxidant assays, phenolic acids and flavonoids determined by HPLC-PDA.

Correlations between the antioxidant assays and total phenolic acids and flavonoids was performed with factor analysis and Pearson’s correlation test (Figure 1 and Table 5). The total phenolic acids and flavonoids were calculated with summation of the proposed compounds based on the HPLC-PDA quantification to give an idea of the general correlations between the overall phenolic compounds and the antioxidant tests. The polyphenols that were not detected in the HPLC-PDA were not considered due to their low concentrations. 

Here, 77.5% variability of the initial data was kept by the first two factors. It is observed that the three antioxidant assays, DPPH, ABTS and FRAP were strongly correlated with each other, in which the Pearson’s correlation coefficient r = 0.934 (*p* < 0.01) were found between ABTS and DPPH assays. This result is in agreement with previous studies [34,39]. Both ABTS and DPPH assays detected free radical scavenging activity of the samples. The ABTS assay is applicable to both hydrophilic and lipophilic antioxidant systems, while DPPH is only applicable to hydrophobic systems due to using a radical dissolved in organic media [40]. The strong correlation between these two parameters in this study indicated that the polyphenols that contributed to the free radical scavenging activity were similar compounds with comparable hydrophilicity. The results also verified that in most of the plant extracts, the results determined by ABTS were higher than those obtained from DPPH assay [34]. FRAP was highly correlated (*p* < 0.01) with both DPPH and ABTS with positive correlation coefficient of r = 0.979 and 0.869 respectively. FRAP tested the reducing capability measured by the ferric ions. The high correlation of FRAP with DPPH and ABTS suggested that the compounds present in the plant exacts capable of scavenge DPPH and ABTS free radicals could also reduce ferric ions. It indicated that it was the reducing activity of the polyphenols that dictated the extent of free radical scavenging, as not all the reducing compounds are antioxidants. The high correlations of FRAP and antioxidant activity assays were previously reported by Pulido, et al. [41] and Vasco, et al. [42]. 

A high positive correlation was found between TPC and the antioxidant parameters with correlation coefficient r > 0.8 (*p* < 0.01) (Table 5). Folin reagents react to polyphenols and any other reducing substance present in the sample. The strong correlations suggested that polyphenols in the plant extracts were the major contributors to the reducibility of the plant extracts. On the other hand, poor positive correlations of TFC, tannin content and antioxidant activities were found, and is likely explained by the TFC and tannin assays only targeting specific flavonoids. The TFC method that involves reaction with aluminum chloride is selective only for flavonols and flavone luteolin, namely the hydroxyl groups at C-3 of C ring, and both C-3 and C-4 of the B ring of the basic structure is necessary [43]. For the tannin assay, only a narrow range of flavanols were specified. The poor correlations indicated that among the selected plant extracts, the contributions from flavonoids to the antioxidant activities were limited. 

The HPLC detected phenolic acids were strongly correlated (*p* < 0.01) with the antioxidant measures (ABTS, FRAP and DPPH) with r = 0.740, 0.803 and 0.711 respectively, suggesting that within the selected samples in this study, the phenolic acids significantly contributed to the antioxidant activities. Although the reducing ends of phenolic acids are fewer than those found in flavonoids, it has been reported that synergism between phenolic acids can occur during the performance of the antioxidant assays [36]. Flavonoids were found to be closely correlated with tannin content (r = 0.831, *p* < 0.01) while less correlated with other parameters, which was consistent with the poor correlations of the antioxidant parameters with TFC and tannin content. The correlation coefficient between the overall flavonoids and TFC was also small (r = 0.386), and this result can be explained by that among the selected targeted flavonoids, tannins accounted for a large percentage. Catechin, epicatechin and epicatechin gallate are tannins with a high proportion of the total flavonoids of the samples, especially for the samples with high total flavonoids content such as blueberry and strawberry. Other flavonoids, such as myricetin, luteolin, apigenin, and anthocyanins are also flavonoids commonly existing in plant foods but not analysed in this study. The presence and activity of these compounds may account for the low correlation between the flavonoids quantified by HPLC and the TFC result. 

## 4. Conclusions

The antioxidant properties of 16 fruits and vegetables were studied in a high-throughput modification of established methods with quantification of individual phenolic acids and the flavonoids determined by separation and spectroscopy. Mango and blueberry had significantly higher antioxidant activities compared to other plant food samples tested. Eighteen phenolic acids and flavonoids were analysed using HPLC-PDA and verified by LC-ESI-QTOF/MS. Most of the phenolic compounds were accurately detected by the HPLC-PDA, whereas some polyphenols such as p-coumaric acid, p-hydroxybenzoic acid, protocatechuic acid, ferulic acid and caffeic acid with low concentrations were mis-detected in the HPLC-PDA but correctly identified in the LC-ESI-QTOF/MS. Factor analysis showed high correlations between ABTS, DPPH and FRAP, implying the antioxidant compounds in the samples were capable of scavenging the DPPH and ABTS free radicals as well as the reducing ferric ions. High correlations found between TPC and the antioxidant parameters suggested the bioactive polyphenols significantly contributed to the reducibility in the plant foods tested. The overall phenolic acids and flavonoids detected by HPLC-PDA were highly correlated with the antioxidant parameters and tannins contents respectively, indicating among the selected plant foods, phenolic acids contributed more to the antioxidant activities while the selected flavonoids contributed more to the tannin content. Developing these assays in high-throughput mode allows more fruits and vegetables of different origins to be analysed. We were able to correlate these assays with HPLC and LC-MS with different detection strategies to understand both composition and functional response in fruits and vegetables. Robust methods and correlations will help scientists understand the structure and function of food and contribute to a healthy food supply.

## Figures and Tables

**Table 1:** Antioxidant potential of selected fruits and vegetables.

Samples	TPC ^1^	TFC ^2^	Tannin ^3^	DPPH ^4^	ABTS ^4^	FRAP ^4^
mango	2.13 ± 0.10 ^a^	0.34 ± 0.04 ^b, c^	0.09 ± 0.02 ^c^	2.34 ± 0.00 ^a^	3.05 ± 0.13 ^a^	3.20 ± 0.05 ^a^
blueberry	2.08 ± 0.06 ^a^	0.46 ± 0.02 ^a^	2.35 ± 0.49 ^a^	1.36 ± 0.03 ^c^	1.60 ± 0.05 ^c^	2.39 ± 0.09 ^b^
strawberry	1.78 ± 0.09 ^b^	0.25 ± 0.02 ^e, f, g^	0.28 ± 0.10 ^c^	1.58 ± 0.06 ^b^	2.23 ± 0.17 ^b^	2.41 ± 0.05 ^b^
black carrot	1.38 ± 0.08 ^c^	0.27 ± 0.03 ^e, f g^	0.22 ± 0.14 ^c^	0.93 ± 0.04 ^d^	0.88 ± 0.04 ^e, f^	1.44 ± 0.05 ^c^
raspberry	1.29 ± 0.03 ^c^	0.17 ± 0.00 ^h, i^	-	1.63 ± 0.02 ^b^	1.83 ± 0.05 ^c^	2.32 ± 0.09 ^b^
grapes	1.26 ± 0.05 ^c^	0.31 ± 0.01 ^c, d, e^	0.19 ± 0.02 ^c^	1.07 ± 0.17 ^d^	1.35 ± 0.11 ^d^	1.36 ± 0.02 ^c^
garlic	1.06 ± 0.00 ^d^	0.12 ± 0.00 ^i^	1.09 ± 0.01 ^b^	0.13 ± 0.00 ^h, i^	0.35 ± 0.00 ^g^	0.06 ± 0.00 ^h, i^
ginger	0.95 ± 0.03 ^d, e^	0.15 ± 0.00 ^i^	0.37 ± 0.00 ^c^	0.21 ± 0.00 ^g, h^	1.09 ± 0.00 ^e^	0.04 ± 0.00 ^i^
onion	1.02 ± 0.00 ^d, e^	0.39 ± 0.00 ^b^	0.02 ± 0.00 ^c^	0.17 ± 0.00 ^h^	0.85 ± 0.01 ^f^	0.05 ± 0.00 ^i^
cherry	0.89 ± 0.07 ^e, f^	0.34 ± 0.01 ^b, c^	-	0.51 ± 0.02 ^e^	0.39 ± 0.17 ^g^	0.86 ± 0.04 ^d^
plum	0.76 ± 0.07 ^f^	0.28 ± 0.01 ^d, e, f^	-	0.40 ± 0.06 ^e, f^	0.44 ± 0.06 ^g^	0.76 ± 0.09 ^d, e^
apple	0.59 ± 0.04 ^g^	0.33 ± 0.02 ^c, d^	-	0.33 ± 0.02 ^f, g^	0.08 ± 0.02 ^h^	0.63 ± 0.02 ^e, f^
papaya	0.50 ± 0.04 ^g, h^	0.31 ± 0.01 ^c, d, e^	-	0.41 ± 0.01 ^e, f^	0.58 ± 0.08 ^g^	0.75 ± 0.02 ^d, e^
peach	0.38 ± 0.03 ^h^	0.24 ± 0.02 ^f, g^	-	0.15 ± 0.00 ^h, i^	-	0.50 ± 0.01 ^f^
pear	0.34 ± 0.02 ^h, i^	0.25 ± 0.03 ^f, g^	0.08 ± 0.02 ^c^	-	0.09 ± 0.01^h^	0.33 ± 0.01 ^g^
apricot	0.19 ± 0.01 ^I, j^	0.22 ± 0.01 ^g, h^	-	-	-	0.19 ± 0.03 ^h^

All values are expressed as mg/g sample ± standard deviation of three independent measurements. ^1^ Total phenolic content is expressed as mg of gallic acid equivalent (GAE)/g of sample. ^2^ Total flavonoid content is expressed as mg of quercetin equivalents (QE)/g of sample. ^3^ Total tannins content is expressed as mg of catechin equivalents (CE)/g of sample. ^4^ Antioxidant activities are expressed as mg of ascorbic acid equivalent (AAE)/g of the sample. Different letters indicate significant differences at *p* < 0.05.

**Table 2:** Polyphenolic contents of different fruits and vegetables quantified using HPLC-PDA.

**No.**	**Compounds**	**RT**	**Mango**	**Blueberry**	**Strawberry**	**Black carrot**	**Raspberry**	**Grapes**	**Garlic**	**Ginger**
1	gallic acid	6.836	83.2 ± 1.44 ^a^	0.02 ± 0.01 ^e^	3.11 ± 0.71 ^d^	-	5.80 ± 1.05 ^c^	0.61 ± 0.08 ^e^	-	-
2	protocatechuic acid	12.569	23.0 ± 5.32 ^a^	5.22 ± 0.09 ^b^	4.30 ± 0.07 ^b^	-	2.78 ± 0.10 ^c^	1.77 ± 0.47 ^d^	2.39 ± 0.19 ^c^	1.77 ± 0.14 ^d^
3	caftaric acid	13.774	-	4.71 ± 0.05 ^b^	5.05 ± 0.08 ^a^	4.75 ± 0.50 ^b^	4.37 ± 0.18 ^b^	4.61 ± 0.08 ^b^	-	-
5	chlorogenic acid	20.24	30.6 ± 1.23 ^a^	7.12 ± 0.51 ^e^	9.70 ± 1.97 ^e^	3.61 ± 0.30 ^c, d^	3.76 ± 0.47 ^f^	12.7 ± 2.02 ^b^	-	-
6	p-hydroxybenzoic acid	20.579	1.40 ± 0.07 ^c^	-	2.80 ± 0.25 ^b^	-	-	-	-	-
7	caffeic acid	25.001	0.24 ± 0.13 ^e^	32.3 ± 0.79 ^a^	0.02 ± 0.46 ^e, f^	2.74 ± 0.12 ^b^	3.73 ± 1.70 ^c^	0.16 ± 0.39 ^e^	-	-
8	syringic acid	26.326	-	7.16 ± 0.38 ^a^	-	-	7.96 ± 1.51 ^a^	-	-	-
10	coumaric acid	34.455	-	7.11 ± 0.18 ^a^	-	0.22 ± 0.07 ^b^	-	-	-	-
12	ferulic acid	39.823	-	2.98 ± 0.52 ^a^	0.06 ± 0.84 ^b^	-	0.12 ± 0.31 ^b^	-	-	-
	**Overall Phenolic Acids**		139 ± 52.8 ^A^	66.6 ± 20.2 ^A, B^	25.1 ± 7.45 ^B^	30.5 ± 9.30 ^B^	28.5 ± 8.18 ^B^	19.8 ± 6.59 ^B^	2.39 ± 0.97 ^B^	1.77 ± 0.72 ^B^
4	catechin	19.704	4.32 ± 0.64 ^c^	81.8 ± 9.17 ^a^	2.51 ± 0.05 ^d^	0.41 ± 0.02 ^d^	-	1.44 ± 0.09 ^e^	0.69 ± 0.26 ^f^	-
9	epicatechin	26.739	-	9.25 ± 0.15 ^a^	6.80 ± 2.20 ^b^	-	2.94 ± 1.25 ^c^	2.02 ± 1.17 ^d^	2.72 ± 0.32 ^d^	-
11	epicatechin gallate	38.015	0.51 ± 0.40 ^b, c^	0.48 ± 0.52 ^c^	0.45 ± 0.32 ^c^	-	-	0.29 ± 0.30 ^d^	0.38 ± 0.24 ^c^	0.27 ± 0.07 ^d^
13	quercetin-3-galactoside	40.134	0.18 ± 0.76 ^c^	0.19 ± 0.09 ^c^	0.35 ± 0.49 ^c^	-	-	-	-	-
14	quercetin-3-glucuronide	40.659	3.00 ± 0.86 ^a^	1.76 ± 0.12 ^b^	3.35 ± 1.58 ^a^	0.53 ± 0.12 ^b, c^	0.54 ± 0.75 ^c^	3.11 ± 1.54 ^a^	-	-
15	quercetin-3-glucoside	45.172	0.61 ± 1.49 ^c^	2.38 ± 0.35 ^b^	0.20 ± 0.48 ^d^	0.14 ± 0.17 ^d^	0.10 ± 0.32 ^e^	0.36 ± 0.48 ^c^	0.16 ± 0.39 ^d, e^	-
16	kaempferol-3-glucoside	47.111	2.98 ± 0.73 ^b^	5.45 ± 0.24 ^a^	1.04 ± 0.28 ^c^	0.60 ± 0.11 ^c^	0.30 ± 0.45 ^e, f^	0.68 ± 1.2 ^d, e^	-	-
17	quercetin	70.098	-	-	19.0 ± 2.20 ^a^	2.34 ± 0.12 ^b^	-	-	2.36 ± 0.16 ^b^	-
18	kaempferol	80.347	7.51 ± 0.07 ^b^	5.17 ± 0.04 ^b^	6.13 ± 0.52 ^b^	-	-	5.35 ± 0.59 ^b^	11.6 ± 1.36 ^b^	57.1 ± 5.05 ^a^
	**Overall Flavonoids**		19.1 ± 2.58 ^A^	107 ± 26.4 ^A^	39.8 ± 6.00 ^A^	7.28 ± 1.04 ^A^	3.89 ± 0.96 ^A^	13.2 ± 1.79 ^A^	14.6 ± 2.71 ^A^	57.4 ± 19.0 ^A^
**No.**	**Compounds**	**RT**	**Onion**	**Cherry**	**Plum**	**Apple**	**Papaya**	**Peach**	**Pear**	**Apricot**
1	gallic acid	6.836	-	-	-	0.51 ± 0.20 ^e^	-	-	11.2 ± 1.25 ^b^	-
2	protocatechuic acid	12.569	1.77 ± 0.54 ^d^	3.28 ± 0.87 ^b, c^	-	-	-	-	-	-
3	caftaric acid	13.774	-	-	-	-	5.29 ± 1.00 ^a^	-	-	-
5	chlorogenic acid	20.240	-	18.2 ± 0.19 ^a^	11.4 ± 1.72 ^d^	1.14 ± 0.35 ^g^	-	2.18 ± 0.84 ^g^	9.33 ± 2.09 ^c^	1.62 ± 0.36 ^g^
6	p-hydroxybenzoic acid	20.579	-	-	-	-	-	11.0 ± 0.39 ^a^	-	-
7	caffeic acid	25.001	-	-	-	0.84 ± 0.38 ^e^	-	1.82 ± 1.17 ^d^	0.01 ± 0.14 ^f^	-
8	syringic acid	26.326	-	-	-	3.04 ± 0.31 ^b^	-	-	-	-
10	coumaric acid	34.455	-	-	-	-	-	-	-	-
12	ferulic acid	39.823	-	0.15 ± 0.28 ^b^	-	2.58 ± 1.74 ^a^	0.01 ± 0.01 ^c^	-	-	-
	**Overall phenolic Acids**		1.77 ± 0.72 ^B^	21.6 ± 8.00 ^B^	11.4 ± 4.60 ^B^	8.10 ± 2.45 ^B^	5.30 ± 2.14 ^B^	15.0 ± 5.22 ^B^	20.5 ± 6.92 ^B^	1.62 ± 0.66 ^B^
4	catechin	19.704	8.01 ± 0.96 ^b^	-	1.26 ± 1.08 ^e, f^	-	-	-	1.34 ± 1.15 ^e, f^	-
9	epicatechin	26.739	0.51 ± 0.21 ^f^	0.23 ± 0.15 ^f^	0.45 ± 0.15 ^f^	0.30 ± 1.11 ^f^	-	-	1.01 ± 1.30 ^e^	0.01 ± 0.12 ^g^
11	epicatechin gallate	38.015	-	0.29 ± 0.39 ^c, d^	0.44 ± 0.41 ^c^	0.69 ± 0.80 ^b^	-	1.02 ± 0.86 ^a^	0.52 ± 0.80 ^b, c^	0.67 ± 1.04 ^b^
13	quercetin-3-galactoside	40.134	-	-	-	4.08 ± 0.36 ^a^	-	1.68 ± 0.99 ^b^	0.14 ± 0.49 ^c^	-
14	quercetin-3-glucuronide	40.659	1.63 ± 1.41 ^b^	0.41 ± 0.17 ^c^	0.76 ± 1.08 ^b, c^	1.27 ± 2.23 ^b, c^	0.43 ± 0.16 ^c^	-	-	0.44 ± 0.21 ^c^
15	quercetin-3-glucoside	45.172	0.14 ± 0.12 ^e^	0.23 ± 0.23 ^c, d^	0.47 ± 0.66 ^c^	3.36 ± 0.19 ^a^	0.21 ± 0.27 ^d^	0.93 ± 1.28 ^c^	0.59 ± 0.57 ^c^	0.25 ± 0.71 ^c, d^
16	kaempferol-3-glucoside	47.111	0.30 ± 0.13 ^f^	0.42 ± 0.41 ^e^	2.73 ± 0.43 ^b^	0.9 ± 1.50 ^c, d^	0.81 ± 1.37 ^d^	0.43 ± 0.18 ^e^	0.91 ± 1.71 ^c, d^	0.30 ± 0.10 ^f^
17	quercetin	70.098	2.66 ± 0.23 ^b^	2.51 ± 0.16 ^b^	2.83 ± 1.17 ^b^	2.94 ± 0.87 ^b^	-	-	-	3.63 ± 0.13 ^b^
18	kaempferol	80.347	6.67 ± 1.25 ^b^	10.0 ± 1.73 ^b^	5.08 ± 0.85 ^b^	-	-	5.77 ± 0.44 ^b^	5.21 ± 0.29 ^b^	5.12 ± 0.49 ^b^
	**Overall Flavonoids**		19.9 ± 3.05 ^A^	14.1 ± 3.26 ^A^	14.0 ± 1.66 ^A^	13.5 ± 1.55 ^A^	1.45 ± 0.28 ^A^	9.82 ± 1.85 ^A^	9.72 ± 1.62 ^A^	10.4 ± 1.88 ^A^

All values are expressed as mg/100 g sample ± standard deviation of three independent measurements. Different letters indicate significant differences at *p* < 0.05. No., the order of the retention time; RT, retention time (min).

**Table 3:** Identification of targeted phenolic compounds by LC-ESI-QTOF/MS.

No.	Compounds	Molecular Formula	RT (min)	Mode of Ionization	Molecular Weight	Theoretical (*m*/*z*)	Observed (*m*/*z*)	Mass Error (ppm)	Sample *
1	gallic acid	C_7_H_6_O_5_	6.836	ESI - / [M − H]^−^	170.0215	169.0142	169.0134	−4.73	grapes
2	protocatechuic acid	C_7_H_6_O_4_	12.569	ESI - / [M − H]^−^	154.0266	153.0193	153.0197	2.61	ginger
3	caftaric acid	C_13_H_12_O_9_	13.774	ESI - / [M − H]^−^	312.0472	311.0399	311.0379	−6.43	papaya
4	catechin	C_15_H_14_O_6_	19.704	ESI - / [M − H]^−^	290.0790	289.0717	289.0704	−4.50	garlic
5	chlorogenic acid	C_16_H_18_O_9_	20.207	ESI - / [M − H]^−^	354.0951	353.0878	353.0868	−2.83	blueberry
6	p-hydroxybenzoic acid	C_7_H_6_O_3_	20.579	ESI - / [M − H]^−^	138.0317	137.0244	137.0237	−5.11	mango
7	caffeic acid	C_9_H_8_O_4_	25.001	ESI - / [M − H]^−^	180.0423	179.0350	179.0341	−5.03	strawberry
8	syringic acid	C_9_H_10_O_5_	26.326	ESI - / [M − H]^−^	198.0528	197.0455	197.0441	−7.10	raspberry
9	epicatechin	C_15_H_14_O_6_	26.739	ESI - / [M − H]^−^	290.0769	289.0696	289.0699	1.04	pear
10	p-coumaric acid	C_9_H_8_O_3_	34.455	ESI - / [M − H]^−^	164.0473	163.0400	163.0393	−4.29	black carrot
11	epicatechin gallate	C_22_H_18_O_10_	38.015	ESI - / [M − H]^−^	442.0900	441.0827	441.0847	4.53	apricot
12	ferulic acid	C_10_H_10_O_4_	39.823	ESI - / [M − H]^−^	194.0579	193.0506	193.0496	−5.18	cherry
13	quercetin-3-*O*-galactoside	C_21_H_20_O_12_	40.134	ESI - / [M − H]^−^	464.0955	463.0882	463.0850	−6.91	apple
14	quercetin-3-*O*-glucuronide	C_21_H_18_O_13_	40.659	ESI - / [M − H]^−^	478.0747	477.0674	477.0648	−5.45	mango
15	quercetin-3-*O*-glucoside	C_21_H_20_O_12_	45.172	ESI - / [M − H]^−^	464.0955	463.0882	463.0889	1.51	blueberry
16	kaempferol-3-*O*-glucoside	C_21_H_20_O_11_	47.111	ESI - / [M − H]^−^	448.1006	447.0933	447.0932	−0.22	peach
17	quercetin	C_15_H_10_O_7_	70.098	ESI - / [M − H]^−^	302.0427	301.0354	301.0329	−8.30	onion
18	kaempferol	C_15_H_10_O_6_	80.347	ESI - / [M − H]^−^	286.0477	285.0404	285.0395	−3.16	plum

* Example sample used for the LC-ESI-MS/QTOF parameters gathering for each polyphenol compound. Molecular formula, retention time (RT), molecular weight, theoretical and observed *m*/*z*, mass error (ppm) and the example sample for each phenolic compound.

**Table 4:** Polyphenol compounds detected and identified in LC-ESI-QTOF/MS (only) for each plant sample.

Samples	Compounds	Molecular Formula	RT (min)	Mode of Ionization	Molecular Weight	Theoretical (*m*/*z*)	Observed (*m*/*z*)	Mass Error (ppm)
mango	syringic acid	C_9_H_10_O_5_	31.541	ESI - / [M − H]^−^	198.0528	197.0455	197.0448	−3.55
	ferulic acid	C_10_H_10_O_4_	38.471	ESI - / [M − H]^−^	194.0579	193.0506	193.0496	−5.18
strawberry	p-coumaric acid	C_9_H_8_O_3_	33.499	ESI - / [M − H]^−^	164.0473	163.0400	163.0393	−4.29
black carrot	ferulic acid	C_10_H_10_O_4_	41.611	ESI - / [M − H]^−^	194.0579	193.0506	193.0494	−6.22
raspberry	p-hydroxybenzoic acid	C_7_H_6_O_3_	20.332	ESI - / [M − H]^−^	138.0317	137.0244	137.0239	−3.65
	p-coumaric acid	C_9_H_8_O_3_	34.745	ESI - / [M − H]^−^	164.0473	163.0400	163.0392	−4.91
garlic	chlorogenic acid	C_16_H_18_O_9_	20.430	ESI - / [M − H]^−^	354.0951	353.0878	353.0863	−4.25
	caffeic acid	C_9_H_8_O_4_	25.218	ESI - / [M − H]^−^	180.0423	179.0350	179.0357	3.91
	p-coumaric acid	C_9_H_8_O_3_	35.288	ESI - / [M − H]^−^	164.0473	163.0400	163.0407	4.29
onion	p-hydroxybenzoic acid	C_7_H_6_O_3_	21.608	ESI - / [M − H]^−^	138.0317	137.0244	137.0242	−1.46
	p-coumaric acid	C_9_H_8_O_3_	35.256	ESI - / [M − H]^−^	164.0473	163.0400	163.0393	−4.29
cherry	caffeic acid	C_9_H_8_O_4_	24.932	ESI - / [M − H]^−^	180.0423	179.0350	179.0346	−2.23
	p-coumaric acid	C_9_H_8_O_3_	37.984	ESI - / [M − H]^−^	164.0473	163.0400	163.0393	−4.29
plum	protocatechuic acid	C_7_H_6_O_4_	12.179	ESI - / [M − H]^−^	154.0266	153.0193	153.0190	−1.96
	p-coumaric acid	C_9_H_8_O_3_	38.180	ESI - / [M − H]^−^	164.0473	163.0400	163.0402	1.23
apple	protocatechuic acid	C_7_H_6_O_4_	12.511	ESI - / [M − H]^−^	154.0266	153.0193	153.0181	−7.84
	p-coumaric acid	C_9_H_8_O_3_	35.473	ESI - / [M − H]^−^	164.0473	163.0400	163.0394	−3.68
peach	protocatechuic acid	C_7_H_6_O_4_	12.165	ESI - / [M − H]^−^	154.0266	153.0193	153.0183	−6.54
papaya	protocatechuic acid	C_7_H_6_O_4_	14.242	ESI - / [M − H]^−^	154.0266	153.0193	153.0192	−0.65
pear	syringic acid	C_9_H_10_O_5_	31.529	ESI - / [M − H]^−^	198.0528	197.0455	197.0441	−7.10
	ferulic acid	C_10_H_10_O_4_	38.508	ESI - / [M − H]^−^	194.0579	193.0506	193.0492	−7.25
apricot	p-hydroxybenzoic acid	C_7_H_6_O_3_	20.671	ESI - / [M − H]^−^	138.0317	137.0244	137.0246	1.46
	p-coumaric acid	C_9_H_8_O_3_	35.495	ESI - / [M − H]^−^	164.0473	163.0400	163.0397	−1.84
	ferulic acid	C_10_H_10_O_4_	38.512	ESI - / [M − H]^−^	194.0579	193.0506	193.0500	−3.11

**Table 5:** Pearson’s correlation coefficients (r) for the relationships between antioxidant assays and phenolic contents.

Variables	DPPH	ABTS	FRAP	TPC	TFC	Tannin	Phenolic Acids
**ABTS**	0.934 **						
**FRAP**	0.979 **	0.869 **					
**TPC**	0.867 **	0.888 **	0.829 **				
**TFC**	0.265	0.190	0.330	0.341			
**Tannin**	0.183	0.193	0.237	0.514 *	0.275		
**Phenolic acids**	0.796 **	0.740 **	0.803 **	0.710 **	0.399	0.238	
**Flavonoids**	0.241	0.339	0.282	0.537 *	0.386	0.831 **	0.262

** Significant correlation with *p* < 0.01; * Significant correlation with *p* < 0.05.

## Supplementary Materials

The following are available online at https://www.mdpi.com/2076-3921/8/9/405/s1, Figure S1: HPLC-PDA profile of standard mixture of 18 polyphenols at different wavelengths; (**a**) 280 nm; (**b**) 320 nm and (**c**) 370 nm, Figure S2: LC-ESI-QTOF/MS characterisation of polyphenols. (**a**) Base peak chromatogram (BPC) of standard mixture of targeted polyphenols run at negative mode ionisation (-ESI / [M − H]-); (**b**) A extracted ion chromatogram (EIC) of targeted polyphenols showing retention times; (**c**) BPC of a sample (cherry) of targeted polyphenols; (**d**) BPC of a sample (pear) of targeted polyphenols; (**e**) EIC of one of the targeted polyphenols (chlorogenic acid) from blueberry showing retention time and (**f**) Observed m/z of a targeted polyphenol (chlorogenic acid) from blueberry with abundance.
