# Supplementary material for: LC-ESI-QTOF/MS Characterisation of Phenolic Acids and Flavonoids in Polyphenol-Rich Fruits and Vegetables and Their Potential Antioxidant Activities
Source: Antioxidants (Basel). 2019 Sep 17;8(9):405. doi: 10.3390/antiox8090405 (PMC6770053; doi:10.3390/antiox8090405)
Supplement: Supplementary file 1 [file antioxidants-08-00405-s001.pdf]

supplementary

# LC-ESI-QTOF/MS Characterisation of Phenolic Acids and Flavonoids in Polyphenol-Rich Fruits and Vegetables and Their Potential Antioxidant Activities

**Chunhe Gu, Kate Howell, Frank R. Dunshea and Hafiz A. R. Suleria ***

School of Agriculture and Food, Faculty of Veterinary and Agricultural Sciences, The University of Melbourne, Parkville, VIC 3010, Australia; chunheg@student.unimelb.edu.au (C.G.); khowell@unimelb.edu.au (K.H.); fdunshea@unimelb.edu.au (F.R.D); hafiz.suleria@unimelb.edu.au (H.A.R.S)

* Correspondence: hafiz.suleria@unimelb.edu.au; Tel.: +61 470 439 670

Received: 03 August 2019; Accepted: 10 September 2019; Published: date

**Abstract:** Polyphenols are naturally occurring compounds found largely in fruits and vegetables. The antioxidant properties of these polyphenols including total phenolic content (TPC), total flavonoid content (TFC), tannin content, 1,1-diphenyl-2-picrylhydrazyl free radical (DPPH), 2,2′-azinobis-(3-ethylbenzo-thiazoline-6-sulfonic acid) (ABTS) scavenging abilities and ferric ion reducing antioxidant power (FRAP) were measured among sixteen (16) plant foods (mango, blueberry, strawberry, black carrot, raspberry, dark grapes, garlic, ginger, onion, cherry, plum, apple, papaya, peach, pear and apricot) by modifying, standardising and translating existing antioxidant methods using a 96-well plate reader. Eighteen targeted phenolic acids and flavonoids were characterised and quantified using high-performance liquid chromatography-photometric diode array (HPLC-PDA) and verified by modifying an existing method of liquid chromatography coupled with electrospray-ionisation triple quadrupole time-of-flight mass spectrometry (LC-ESI-QTOF/MS). While most of these compounds were accurately detected by the HPLC-PDA at a low concentration, a few polyphenols in low concentrations could be only be characterised using the LC-ESI-QTOF/MS method. Our results showed that mango possessed the highest overall antioxidant activity, phenolic acid and flavonoid content among the selected fruits. Factor analysis (FA) and Pearson's correlation tests showed high correlations among ABTS, DPPH, FRAP and phenolic acids, implying the comparable capabilities of scavenging the DPPH/ABTS free radicals and reducing ferric ions from the antioxidant compounds in the samples. Phenolic acids contributed significantly to the antioxidant activities, and flavonoids contributed more to tannin content based on the correlations. Overall, methods modified and standardized in this study can provide better understanding of high throughput technologies and increase the reliability of antioxidant data of different plant foods.

**Keywords:** Polyphenols; fruits and vegetables; antioxidant activities; phenolic acids; flavonoids; HPLC-PDA; LC-ESI-QTOF/MS

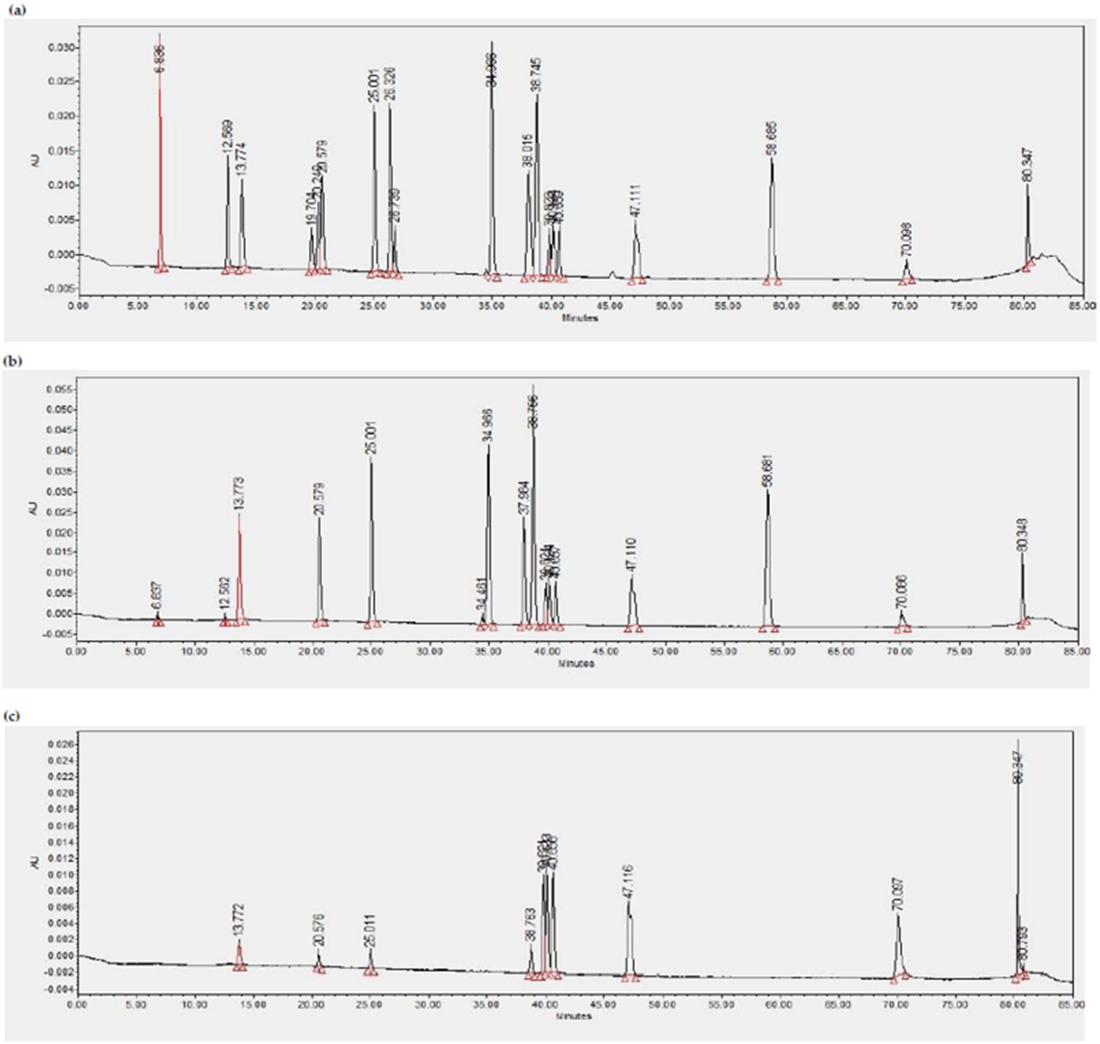


**Figure S1:** HPLC-PDA profile of standard mixture of 18 polyphenols at different wavelengths; (**a**) 280 nm; (**b**) 320 nm and (**c**) 370 nm.

(a)

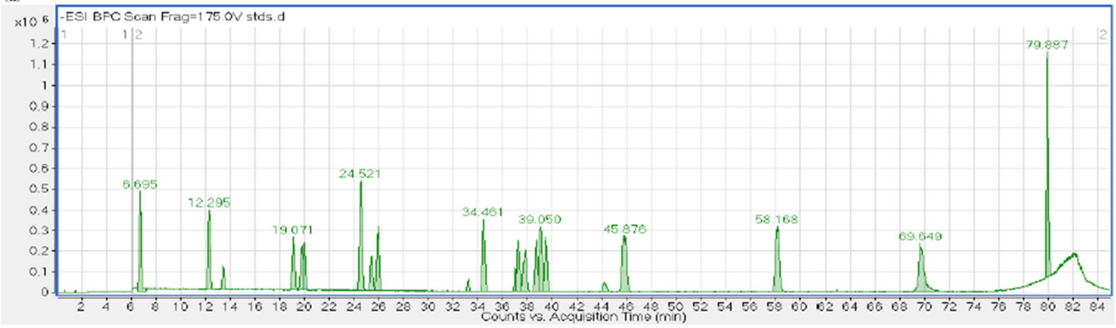
-ESI BPC Scan Frag=175.0V stds.d
x10 6
6.695
12.295
19.071
24.521
34.461
39.050
45.876
58.168
69.649
79.887
Counts vs. Acquisition Time (min)

(b)

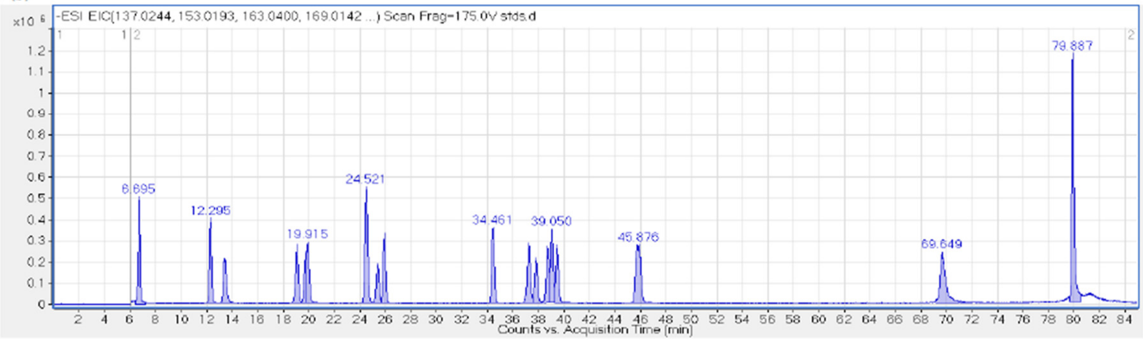
-ESI EIC(137.0244, 153.0193, 163.0400, 169.0142 ...) Scan Frag=175.0V stds.d
x10 6
6.695
12.295
19.915
24.521
34.461
39.050
45.876
69.649
79.887
Counts vs. Acquisition Time (min)

(c)

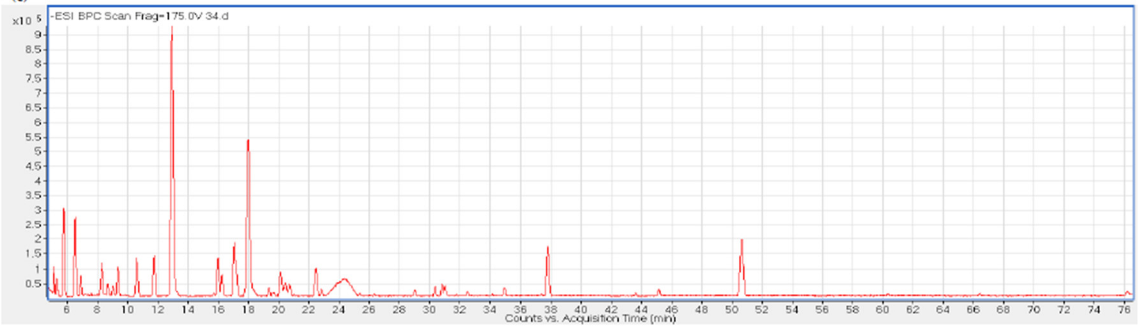
-ESI BPC Scan Frag=175.0V 34.d
x10 5
Counts vs. Acquisition Time (min)

(d)

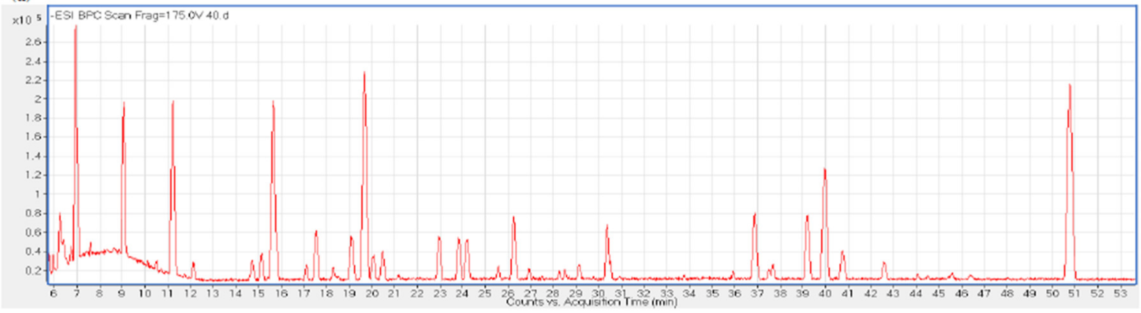
-ESI BPC Scan Frag=175.0V 40.d
x10 5
Counts vs. Acquisition Time (min)

(e)

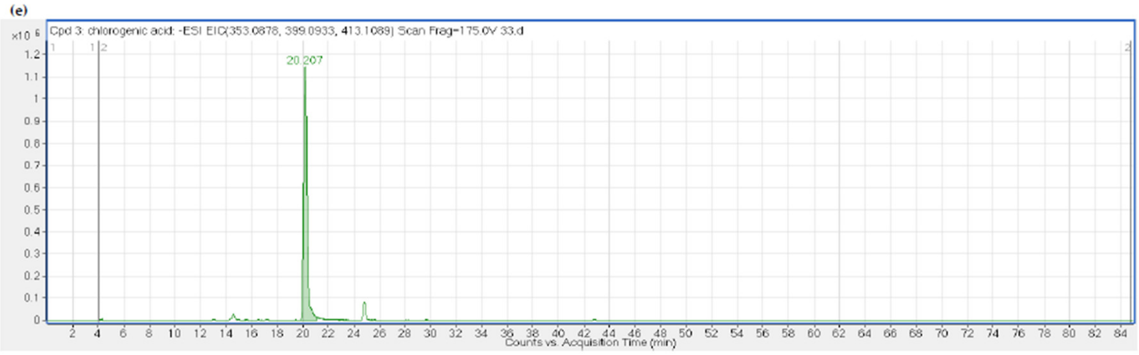


(f)

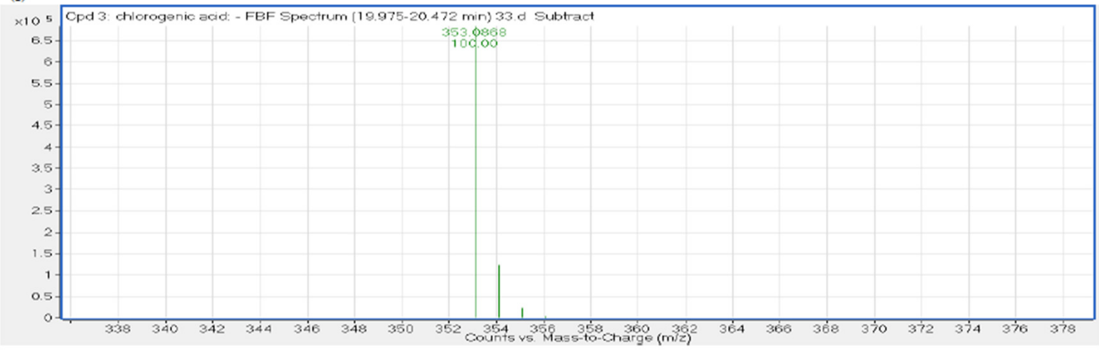


**Figure S2**: LC-ESI-QTOF/MS characterisation of polyphenols. (**a**) Base peak chromatogram (BPC) of standard mixture of targeted polyphenols run at negative mode ionisation (−ESI / [M−H]⁻); (**b**) A extracted ion chromatogram (EIC) of targeted polyphenols showing retention times; (**c**) BPC of a sample (cherry) of targeted polyphenols; (**d**) BPC of a sample (pear) of targeted polyphenols; (**e**) EIC of one of the targeted polyphenols (chlorogenic acid) from blueberry showing retention time and (**f**) Observed m/z of a targeted polyphenol (chlorogenic acid) from blueberry with abundance.
